# The m6A RNA Demethylase ALKBH5 Promotes Radioresistance and Invasion Capability of Glioma Stem Cells

**DOI:** 10.3390/cancers13010040

**Published:** 2020-12-25

**Authors:** Aline Kowalski-Chauvel, Marie Géraldine Lacore, Florent Arnauduc, Caroline Delmas, Christine Toulas, Elizabeth Cohen-Jonathan-Moyal, Catherine Seva

**Affiliations:** 1 INSERM UMR.1037-Cancer Research Center of Toulouse (CRCT)/University Paul Sabatier Toulouse III, 31100 Toulouse, France; aline.chauvel@inserm.fr (A.K.-C.); marie-geraldine.lacore@evotec.com (M.G.L.); florent.arnauduc@inserm.fr (F.A.); Delmas.Caroline@iuct-oncopole.fr (C.D.); toulas.christine@iuct-oncopole.fr (C.T.); moyal.elizabeth@iuct-oncopole.fr (E.C.-J.-M.); 2 IUCT-Oncopole Toulouse, 31000 Tolouse, France

**Keywords:** glioblastomas, radio-resistance, signaling, ALKBH5, cancer stem cells

## Abstract

**Simple Summary:**

Glioblastoma stem cells (GBMSCs), which are particularly radio-resistant and invasive, are responsible for the high recurrence of glioblastoma (GBM). Therefore, there is a real need for a better understanding of the mechanisms involved in these processes and to identify new factors that might be targeted to radiosensitize GBMSC and decrease their invasive capability. Here, we report that the m6A RNA demethylase ALKBH5, which is overexpressed in GBMSCs, promotes their radioresistance by controlling the homologous repair. ALKBH5 was also involved in GBMSC invasion. These data suggest that ALKBH5 inhibition might be a novel approach to radiosensitize GBMSCs and to overcome their invasiveness.

**Abstract:**

Recurrence of GBM is thought to be due to GBMSCs, which are particularly chemo-radioresistant and characterized by a high capacity to invade normal brain. Evidence is emerging that modulation of m6A RNA methylation plays an important role in tumor progression. However, the impact of this mRNA modification in GBM is poorly studied. We used patient-derived GBMSCs to demonstrate that high expression of the RNA demethylase, ALKBH5, increases radioresistance by regulating homologous recombination (HR). In cells downregulated for ALKBH5, we observed a decrease in GBMSC survival after irradiation likely due to a defect in DNA-damage repair. Indeed, we observed a decrease in the expression of several genes involved in the HR, including CHK1 and RAD51, as well as a persistence of γ-H2AX staining after IR. We also demonstrated in this study that ALKBH5 contributes to the aggressiveness of GBM by favoring the invasion of GBMSCs. Indeed, GBMSCs deficient for ALKBH5 exhibited a significant reduced invasion capability relative to control cells. Our data suggest that ALKBH5 is an attractive therapeutic target to overcome radioresistance and invasiveness of GBMSCs.

## 1. Introduction

Glioblastoma (GBM) is the most aggressive primary brain tumor in adults. These tumors exhibit diffuse infiltration into brain tissue, rendering surgical resection difficult and incomplete. GBM are also particularly resistant to adjuvant treatments after surgery. Despite the combination of radiotherapy and chemotherapy, most GBM recurs and median survival remains around 15 months [1]. It is speculated that this high recurrence is due to the presence of glioblastoma stem cells (GBMSCs), which are characterized by their ability of self-renewal, the overexpression of stem cell markers, their pluripotent aptitude to differentiate into neural lineages, and their high tumorigenic potential in vivo [2,3]. GBMSCs are particularly chemo-radioresistant and are also characterized by a high capacity to invade surrounding normal brain [4,5]. Therefore, an understanding of the molecular mechanisms leading to cellular invasion and resistance of GBMSCs is critically needed.

N6-methyladenosine (m6A), methylated at the N6 position of adenosine, is the most abundant modification of mRNA in mammalian cells. Methyltransferases METTL3/METTL14 and their co-factors, also known as m6A “writers”, catalyze the formation of m6A, whereas the demethylases FTO and ALKBH5, named m6A “erasers”, selectively remove m6A modifications. These modifications regulate the splicing, stability, and translation efficiency of specific subsets of mRNA targets [6]. Abnormal expression of m6A regulators has been reported in different cancers and associated in particular with cell proliferation and maintenance of cancer stem cells [7,8,9,10]. However, RNA m6A methylation in cancers is little studied. In GBM, m6A RNA demethylases have been clearly identified as oncogenic factors [11]. Cui et al. showed that inhibition of the m6A RNA demethylase FTO with a specific inhibitor decreased GBMSC tumorigenicity [12]. Su et al. also confirmed that decreasing FTO activity in GBM cell lines led to the inhibition of cell proliferation and viability likely through the regulation of Myc expression [13]. In addition, the m6A RNA demethylase ALKBH5 has been shown to be overexpressed in GBMSCs and to predict poor survival of GBM patients. In this study, ALKBH5 contributed to GBMSC maintenance, proliferation, and tumorigenicity by enhancing the expression of the transcription factor FOXM1 [14]. Recently, the role of ALKBH5 has been confirmed in the proliferation of GBM cell lines via the upregulation of SOX2 expression [15].

GBMSCs are particularly radioresistant, leading to local recurrence that occurs almost systematically. Targeting factors responsible for this radioresistance to increase radiotherapy effects might be an effective approach. Although several targeted therapies have been tested in clinical trials, they are not used to date as standard treatments in GBM to decrease radioresistance [16]. Therefore, it seems important to better understand the mechanisms involved in GBMSC radioresistance to identify new targets.

Although m6A RNA demethylases have been described as oncogenic factors in different cancers, including GBM, their role in the regulation of radiotherapy resistance remains largely unknown. No data are available concerning the involvement of m6A RNA demethylases, ALKBH5, or FTO in radioresistance of GBMSCs. In other cancer types, the role of ALKBH5 in resistance to radiotherapy has never been reported and only one study on cervical cancer has reported the role of the mRNA demethylase FTO on radioresistance [17].

Similarly, to radioresistance, the potential role of m6A RNA demethylases in the invasive capability of GBMSCs is not known. In addition, the involvement of RNA demethylases in cancer cell invasion remains controversial and has been reported by few studies. In breast, lung, and gastric carcinomas, the overexpression of ALKBH5 or FTO have been shown to promote cell invasion [18,19,20,21,22]. On the contrary, m6A RNA methylation could facilitate cell migration in other cancers, including, prostate, pancreas, colon, or hepatic cancers [23,24,25,26]. 

Since ALKBH5 is clearly overexpressed in GBMSC, we focused our study on this m6A RNA demethylase and tested whether the overexpression of ALKBH5 observed in GBMSC derived from human glioblastoma biopsy specimens has a functional role in mediating resistance to radiotherapy and invasion. We also deciphered the possible mechanisms by which this enzyme might regulate these two processes. 

Our data demonstrate that targeting ALKBH5 increases radiosensitization of GBMSCs and represses their invasion capability and suggest that ALKBH5 is an attractive therapeutic target to overcome radioresistance and invasiveness of GBM cancer cells.

## 2. Results

### 2.1. Downregulation of ALKBH5 Gene Expression Radiosensitizes GBMSCs

**Figure 1:**
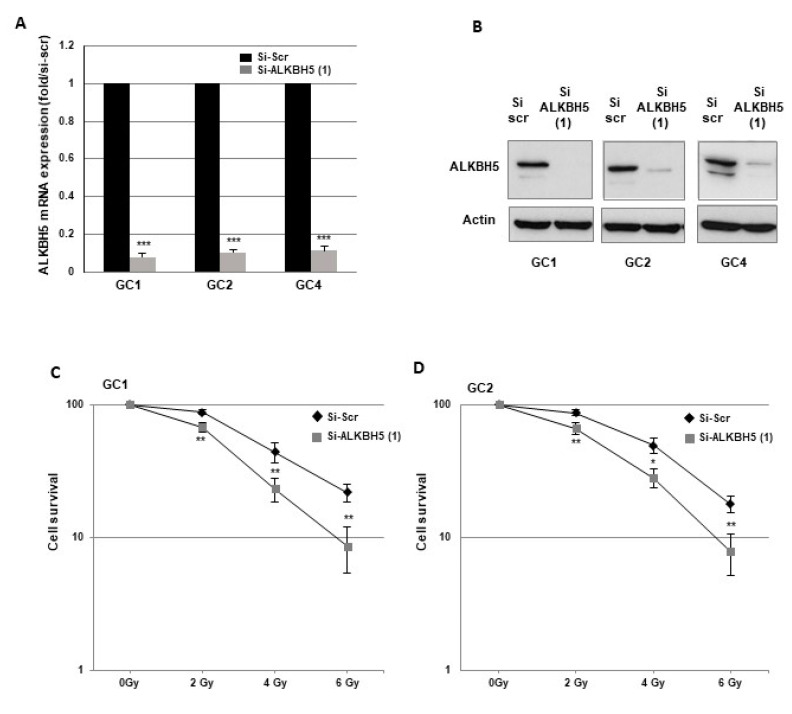

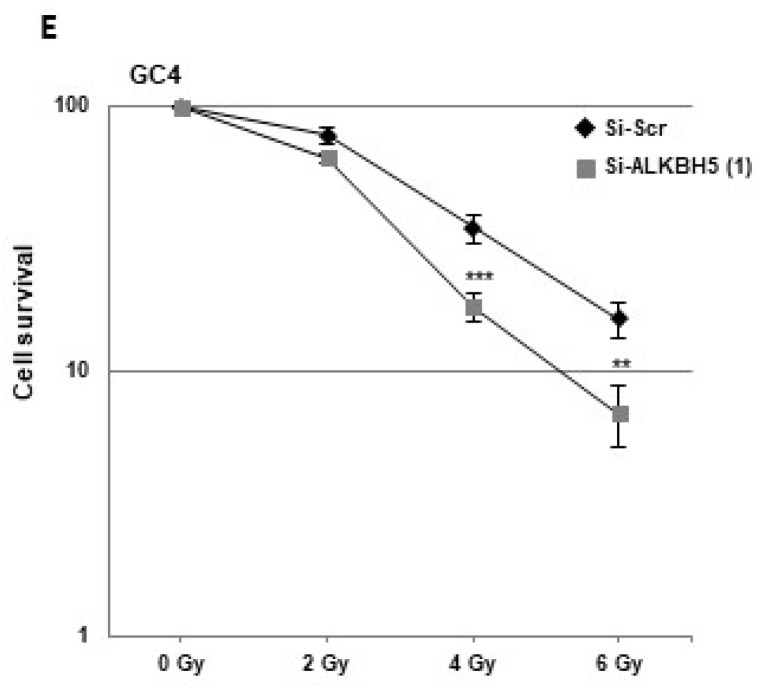
Downregulation of ALKBH5 gene expression radiosensitizes GBMSCs. Primary neurospheres from 3 different patients (GC1, GC2, and GC4) expressing a specific siRNA (si-ALKBH5 (1)) or a scramble control (si-Scr) were used. ALKBH5 expression was analyzed by (**A**) real-time PCR or (**B**) Western blot. Images are representative of three independent experiments. (**C**–**E**) 3-D clonogenic assays were performed as described in “Methods”. Quantifications of 3 experiments are presented as means ±SD. *** *p* < 0.001; ** 0.001 < *p* < 0.01; * 0.01 < *p* < 0.05. Original Western blot gels for (**B**) available in Supplementary Figure S2.

To determine the role of ALKBH5 in radioresistance, we used GBMSCs derived from three human GBM biopsy specimens (GC1, GC2, GC4) cultured as primary neurospheres, and previously characterized [27,28,29,30]. First, we showed that specific ALKBH5 siRNA significantly inhibited mRNA expression compared to a scramble control in the three neurosphere cell lines (Figure 1A). Inhibition was also confirmed at the protein level by Western blot analysis (Figure 1B). 

To determine whether ALKBH5 affects radiation sensitivity, we performed 3-D clonogenic survival assays with increasing doses of irradiation (IR) (Figure 1C–E). The survival fractions after IR were significantly decreased in all GBMSCs transfected with the specific ALKBH5 siRNA compared to the control siRNA, indicating that downregulation of ALKBH5 mRNA expression radiosensitizes GBMSCs derived from human glioblastoma biopsy specimens. 

To assess a potential off-target effect of the ALKBH5 siRNA, we used a second siRNA and showed in GBMSCs a high inhibition of ALKBH5 expression at the mRNA and protein level as well as similar results on clonogenic survival assays under IR (Figure S1A–C). Whereas demethylases, such as ALKBH5, remove N6-methylation of adenosine, the m6A modification is catalyzed by methyltransferases, particularly METTL3 and METTL14. We therefore compared, by real-time PCR, the relative expression of these methyltransferases with that of ALKBH5 in GBMSC. Results in Figure S1D show a 3- to 5-fold higher expression of ALKBH5 compared to MTTL3 and MTTL14 in the GBMSCs used in this study.

### 2.2. Targeting ALKBH5 in GBMSCs Inhibits the Expression of Key DNA Damage Response Genes

**Figure 2:**
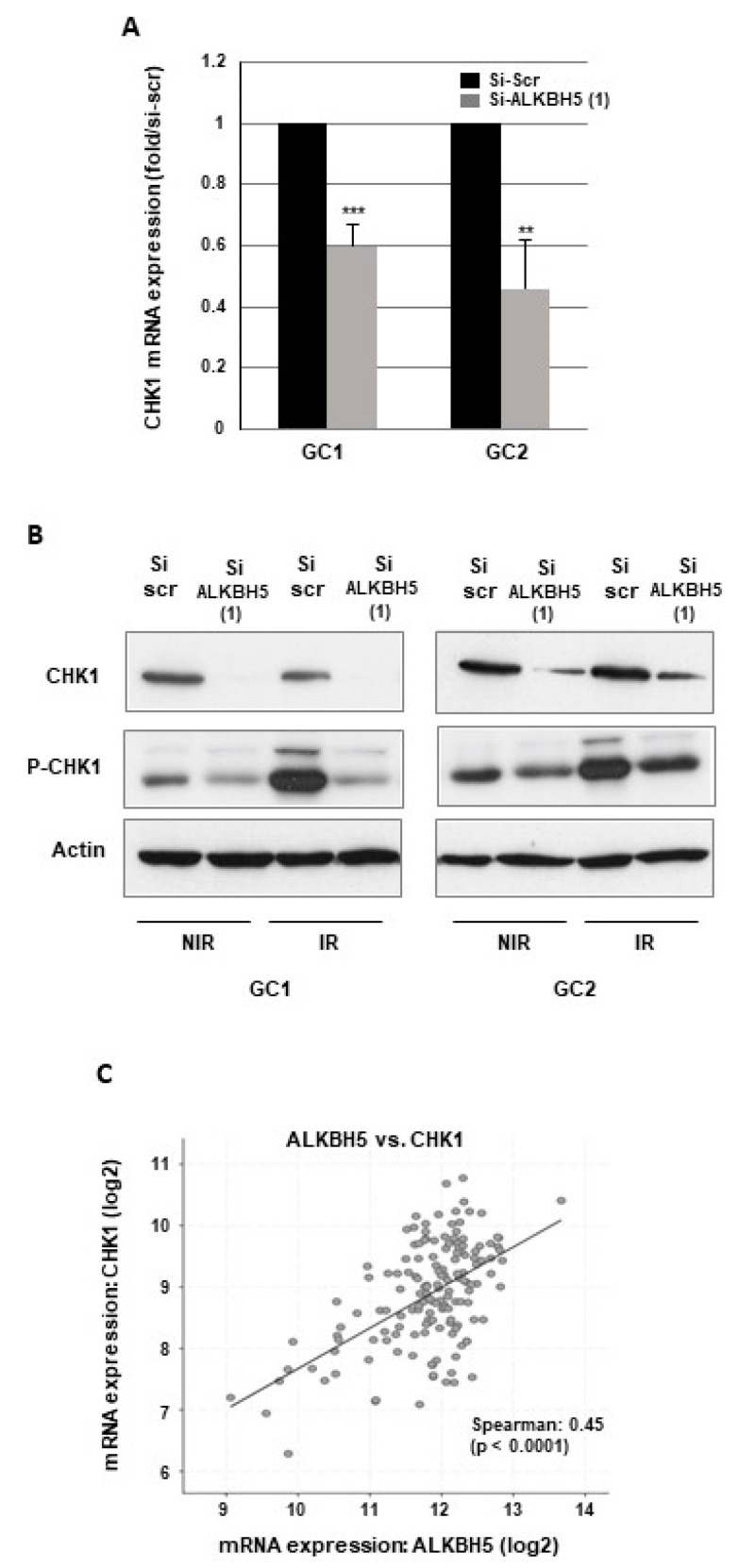
ALKBH5 regulates CHK1 expression in GBMSCs. CHK1 expression and phosphorylation under basal conditions or after irradiation was analyzed by (**A**) real-time PCR or (**B**) Western blot in primary neurospheres from 2 different patients (GC1, GC2) expressing a specific siRNA (si-ALKBH5 (1)) or a scramble control (si-Scr). Quantifications of 3 experiments are presented as means ±SD. *** *p* < 0.001; ** 0.001 < *p* < 0.01; * 0.01 < *p* < 0.05. Images are representative of three independent experiments. (**C**) The correlations between ALKBH5 expression and CHK1 were obtained by the co expression analysis in cBioPortal (https://www.cbioportal.org) using the TCGA database. Values correspond to Spearman’s rank correlation coefficient and its associated *p*-value *p* < 0.001. Original Western blot gels for (**B**) are available in Supplementary Figure S3.

Radioresistance is determined by the ability of cancer cells to repair radiation-induced DNA damages an upregulated DNA damage repair (DDR) has been reported in GBMSCs [31,32,33]. We therefore investigated the role of ALKBH5 in the DDR by evaluating if this m6A RNA demethylase regulates the expression of target genes involved in different DNA repair pathways. In the first steps of the DDR, the checkpoint kinases CHK1 and CHK2 are activated by the effector kinases ATR and ATM, resulting in the initiation of cell cycle checkpoints, cell cycle arrest, and DNA repair [34]. Following ALKBH5 knockout with specific siRNA, we observed a significant inhibition of CHK1 mRNA expression compared to a scramble control (Figure 2A). 

Similar results were obtained with a second ALKBH5 siRNA (Figure S1E). A high inhibition of CHK1, when ALKBH5 is downregulated, was also confirmed at the protein level by Western blot analysis (Figure 2B). In addition, we observed, in response to IR, an increase in CHK1 phosphorylation that was inhibited by ALKBH5 siRNA (Figure 2B), indicating that the activation of CHK1 following IR-induced DNA damage can be overcome by blocking the m6A RNA demethylase. In contrast, blocking ALKBH5 did not affect the expression of CHK2, ATR, or ATM. Since the results in GBMSCs showed that ALKBH5 could regulate CHK1 expression, we then analyzed the correlation between ALKBH5 and CHK1 expression using the glioblastoma database of the Cancer Genome Atlas (TCGA). As shown in Figure 2C, a significant positive correlation was observed between the expression of the two genes.

**Figure 3:**
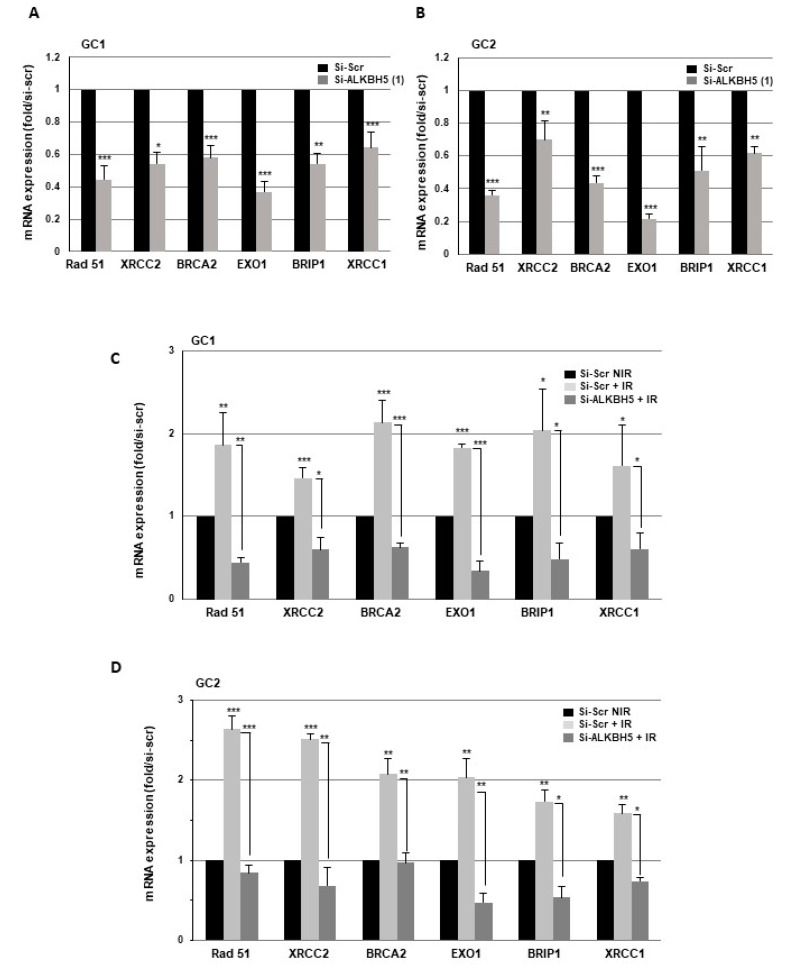
Targeting ALKBH5 in GBMSCs inhibits the expression of key DNA damage response genes. mRNA expressions of key DNA damage response genes under basal conditions (NIR) or after IR were analyzed by real-time PCR in primary neurospheres from 2 different patients (GC1, GC2) expressing a specific siRNA (si-ALKBH5 (1)) or a scramble control (si-Scr). Quantifications of 3 experiments are presented as means ±SD. *** *p* < 0.001; ** 0.001 < *p* < 0.01; * 0.01 < *p* < 0.05.

Cells use two main pathways to repair double-strand breaks (DSBs), homologous recombination repair (HRR) and nonhomologous end joining (NHEJ). We next examined the role of ALKBH5 in the expression of key DNA damage repair players involved in these two pathways. First, we observed in the primary cell lines transfected with specific ALKBH5 siRNA compared to a scramble control a significantly reduced expression of several genes involved in HRR. In particular, Rad51, XRCC2, BRCA2, EXO1, and BRIP1 were downregulated (Figure 3A,B). Similar results were obtained on Rad51 expression with a second ALKBH5 siRNA (Figure S1E). We next analyzed the effect of IR on the expression of these HRR target genes in GBMSCs. As shown in Figure 3C,D, IR significantly increased Rad51, XRCC2, BRCA2, EXO1, and BRIP1 mRNA levels, whereas the induction by radiations was abrogated when ALKBH5 was inhibited by specific siRNA, indicating that blocking this m6A RNA demethylase was able to overcome the upregulation of DDR genes induced by IR. In contrast, the expression of the key genes involved in NHEJ, including Ku70, Ku80, and DNA-PKs, was not affected by ALKBH5 knockdown (data not shown).

**Figure 4:**
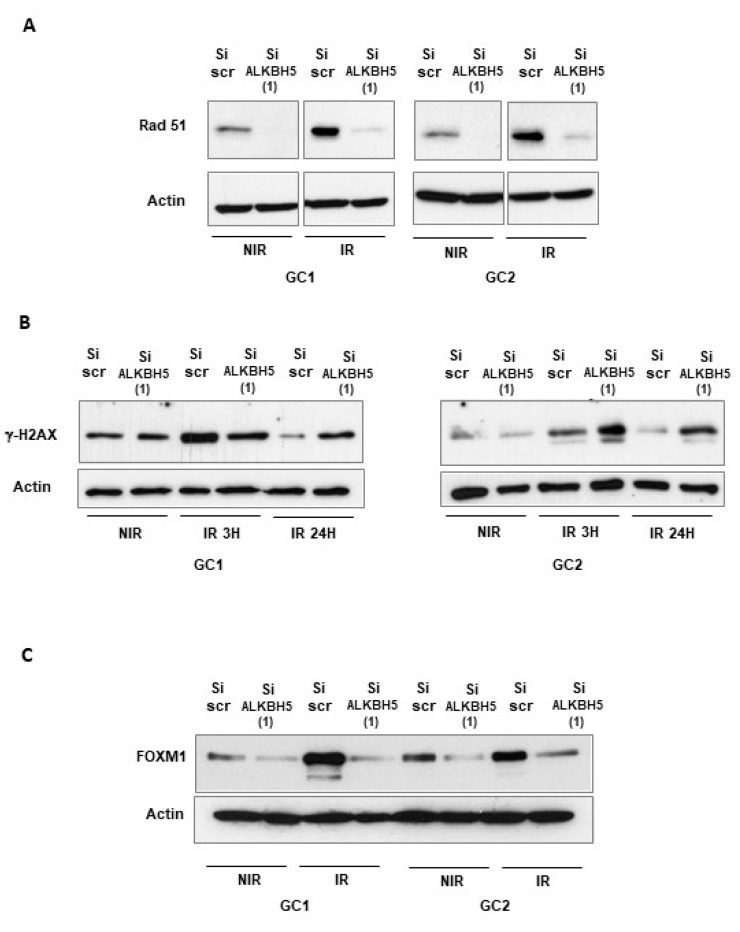
Downregulation of ALKBH5 inhibits the expression of Rad 51 and FOXM1 and decreases DNA repair following IR. Rad 51, FOXM1, and γ-H2AX protein expression, under basal conditions (NIR), or after IR were analyzed by Western blot in primary neurospheres from 2 different patients (GC1, GC2) expressing a specific siRNA (si-ALKBH5 (1)) or a scramble control (si-Scr). Images are representative of three independent experiments. Original Western blot gels for (**A**–**C**) available in Supplementary Figures S4 and S5.

The recombinase Rad51 is the central protein involved in the homologous recombination of DNA during double-strand break repair [35]. Previous reports have confirmed a high expression of Rad51 in GBMSCs and its important role in resistance to radiotherapy [4,33,36,37]. As mentioned above, Rad51 mRNA expression can be regulated by ALKBH5. We then evaluated Rad51 levels by Western blot in GBMSCs following ALKBH5 knockdown. We observed that IR induced an upregulation of Rad51 protein levels, which was attenuated by ALKBH5 inhibition (Figure 4A). Phosphorylation of the Ser-139 residue of the histone H2AX (γ-H2AX) is an early cellular response to DSBs and its quantification is a very sensitive method of detecting DSBs [38]. To confirm that ALKBH5 could affect the repair of IR-induced DSBs, we examined the expression levels of the DSBs marker γ-H2AX by Western blot analysis. As expected, γ-H2AX increased rapidly following IR (3 h) and returned to basal levels at 24 h in control cells, indicating an efficient DDR process in GBMSC neurospheres (Figure 4B). However, in cells transfected with an ALKBH5 siRNA, the DNA damages persisted significantly 24 h post-IR (Figure 4B), indicating that blocking ALKBH5 can delay DDR, improving the radiotherapy efficacy. The transcriptional factor FOXM1 is known to play an essential role in cell cycle, proliferation, DNA replication, and stem cell maintenance [39]. We have recently shown that FOXM1 is a key regulator of GBMSC radioresistance [27]. In addition, Zhang et al. reported the regulation of FOXM1 expression by ALKBH5 [14]. We therefore examined whether the activation of FOXM1 following IR could be regulated by ALKBH5. As shown in Figure 4C, we observed that IR induced an upregulation of FOXM1 protein levels, which was attenuated by ALKBH5 inhibition.

### 2.3. Blocking ALKBH5 Expression Represses Invasion of GBMSCs

**Figure 5:**
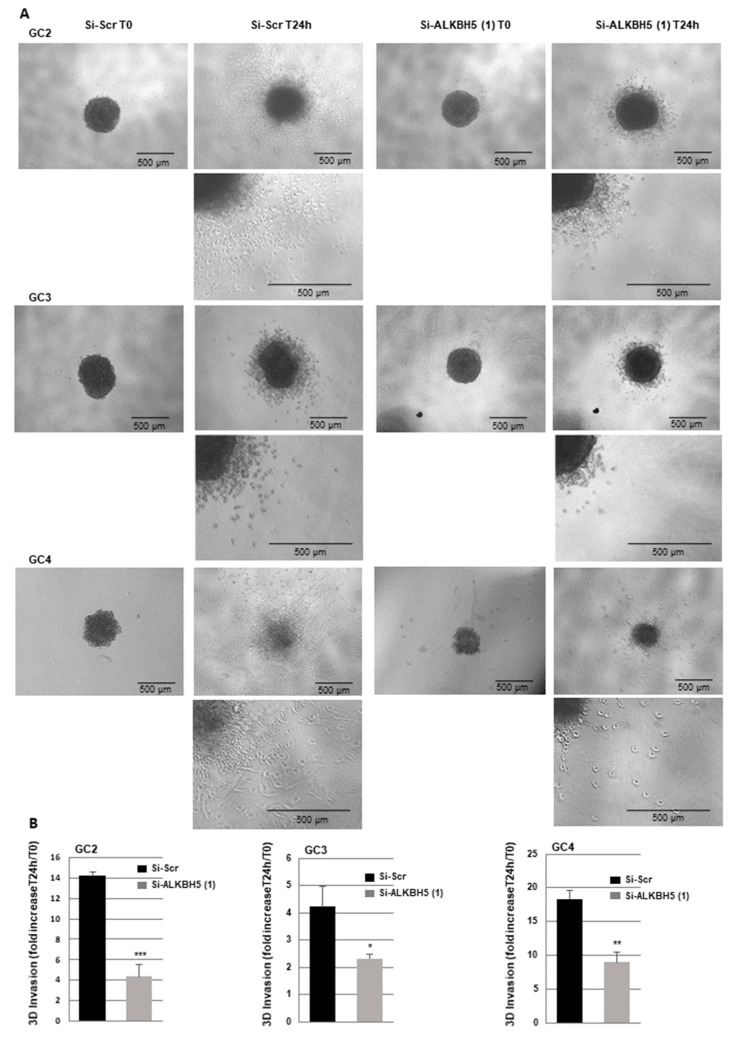
Blocking ALKBH5 expression represses 3-D invasion of GBM spheroid. Primary neurospheres from 3 different patients (GC1, GC2, and GC4) expressing a specific siRNA (si-ALKBH5 (1)) or a scramble control (si-Scr) were used to analyze 3-D invasion as described in the methods. (**A**) Representative micrographs of Matrigel-embedded GBM spheroids were taken just after embedding (T0) or 24 h after invasion into the Matrigel. (**B**) Quantification of tumor cells invasion was performed as described in the methods on 3 independents experiments and presented as means ±SD. *** *p* < 0.001; ** 0.001 < *p* < 0.01.

The role of ALKBH5 in tumor cell invasion remains unclear. To investigate if ALKBH5 could be involved in this process in GBMSCs, we performed 3-D invasion assays in Matrigel, as described in the methods, using invasive primary neurospheres from three different patients in which ALKBH5 was knocked down with a specific siRNA. As shown in Figure 5, all GBMSC spheroids deficient for ALKBH5 exhibited a significant reduced invasion capability relative to control spheroids. 

The transcriptional co-activator Yes-associated protein 1 (YAP1) is known to regulate the expression of target genes involved in the interactions between the extracellular matrix and cancer cells, and plays an important role in cellular invasion [40]. YAP1 is often highly activated in cancer stem cells and recently, Yu et al. demonstrated that YAP1 signaling is required for migration and invasion of GBMSCs [41]. 

**Figure 6:**
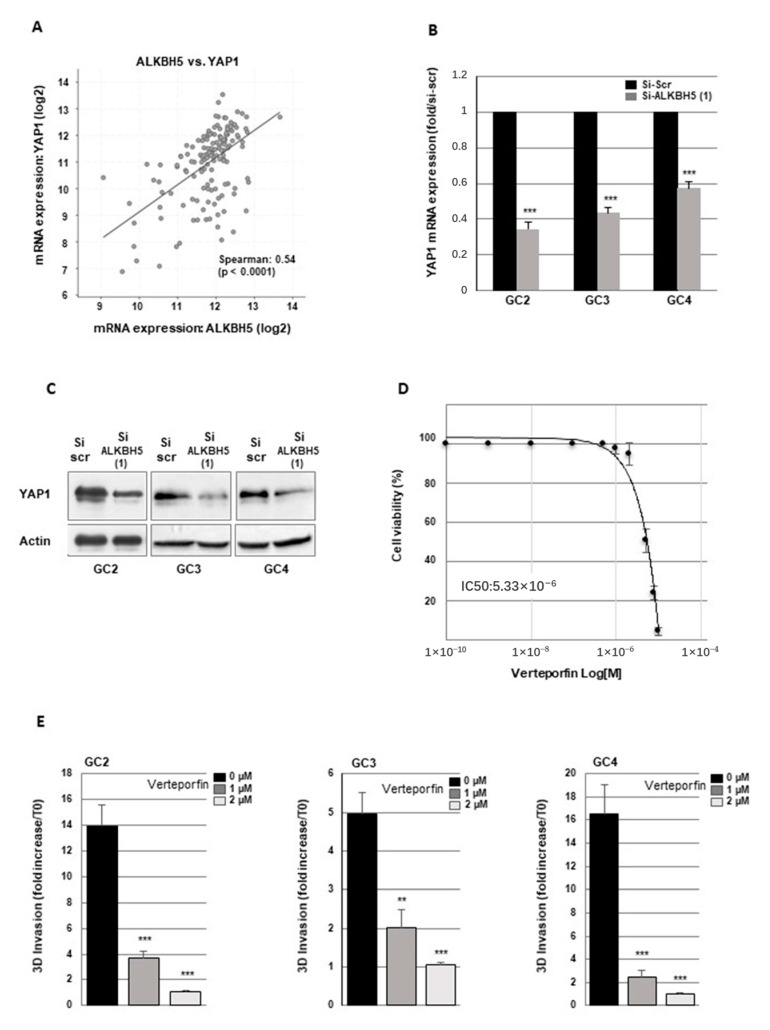
ALKBH5 regulates GBM cells invasion through YAP1 expression. (**A**) The correlations between ALKBH5 expression and YAP1 were obtained by the co-expression analysis in cBioPortal (https://www.cbioportal.org) using the TCGA database. Values correspond to Spearman’s rank correlation coefficient and its associated *p*-value *p* < 0.001. (**B**,**C**) YAP1 expression was analyzed by (**B**) real time PCR or (**C**) western blot in primary neurospheres from 3 different patients (GC2, GC3, GC4) expressing a specific siRNA (si-ALKBH5 (1)) or a scramble control (si-Scr). (**D**) Dose-response curve of verteporfin on cell viability of GC2. (**E**) Quantification of 3-D invasion performed with Image J software from micrographs of Matrigel-embedded GBM spheroids. Three independent experiments and presented as means ±SD. *** *p* < 0.001 ** 0.001 < *p* < 0.01. Original Western blot gels for (**C**) available in Figure S6.

We first analyzed the correlation between ALKBH5 and YAP1 expression using the glioblastoma database of the Cancer Genome Atlas, (TCGA). As shown in Figure 6A, a significant positive correlation was observed between the expression of the two genes. 

We therefore determined if ALKBH5 could regulate YAP1 expression in GBMSCs. Following ALKBH5 knockout in GBMSC with specific siRNA, we observed a significant inhibition of YAP1 mRNA expression compared to a scramble control (Figure 6B). A high inhibition of YAP1, when ALKBH5 is downregulated, was also confirmed at the protein level by Western blot analysis (Figure 6C). Surprisingly, the expression of YAP1 paralog TAZ (transcriptional co-activator with PDZ-binding motif) was not altered by ALKBH5 knockout (data not shown). 

Verteporfin was originally designed as a photosensitizer; however, without photoactivation, it can disrupt the interaction and the transcriptional activity of YAP/TEAD and thus inhibit YAP-mediated effects, leading to reduced YAP1 signaling [42,43]. In addition, verteporfin was previously used in GBM to show the role of YAP1 on cell growth, survival, and resistance to chemotherapy [44,45,46]. First we performed a dose response curve to evaluate the effect of verteporfin on cell viability (Figure 6D). The doses 1 and 2 µM which do not affect cell viability were chosen to assess the impact of verteporfin on cell invasion. Verteporfin was added to the medium of neurospheres before Matrigel solidification. 24 h after Verteporfin treatment at 1 and 2 µM, we observed a significant decrease of Matrigel invasion by GBM cells (Figure 6E). To confirm the specific role of YAP1 in GBMSC, we also used YAP1 siRNA, previously validated in GC2 [28] in the 3D invasion assay and observed a similar decrease in Matrigel invasion by GBMSC (Figure S1F).

### 2.4. Molecular Signatures Associated with High ALKBH5 Expression

In a previous study, Cui et al. reported low levels of m6A RNA modification in GBMSCs [12]. In addition, Zhang et al. showed that blocking ALKBH5 in GBMSCs, using an shRNA strategy, affects m6A global levels [14], suggesting that high expression of ALKBH5 might be more widely associated with general pathways involved in hallmarks of cancers. Analysis of the TCGA database based on ALKBH5 expression (the 25% highest versus 25% lowest expression as described in the methods) revealed the activation of several molecular pathways, including growth factors activity, inflammatory and immune response, and more particularly numerous molecular signatures related to cell motility and adhesion to the extracellular matrix (Table S1).

## 3. Discussion

The m6A mRNAs methylation in cancers is a very recent field of investigation. Methyltransferases METTL3/METTL14 and their co-factors, also known as m6A “writers”, catalyze the formation of m6A whereas the demethylases FTO and ALKBH5, named m6A “erasers”, selectively remove m6A modifications. Evidence is emerging that modulation of m6A RNA methylation plays an important role in different aspects of tumor biology, including growth, tumorigenesis, and self-renewal of cancer stem cells [7,8,9,10]. In brain tumors, the two m6A mRNA demethylases FTO and ALKBH5 have been clearly associated to pro-oncogenic functions, promoting cell proliferation, self-renewal, and the progression of GBMSC-initiated tumors [11,12,13,14,15]. In contrast, the role of the methyltransferases in GBM is more controversial. In accordance with the pro-oncogenic role of demethylases described above, Cui et al. reported a tumor-suppressor role of the methyltransferases METTL3/METTL4 by demonstrating that knocking down these enzymes enhances GBMSC growth and self-renewal [12]. In contrast Visvanathan described a pro-oncogenic role of METTL3 in glioma stem cells [47]. 

The role of ALKBH5 in radioresistance of GBM or other cancer types has never been reported. To our knowledge, this study is the first one demonstrating that this m6A mRNA demethylase is responsible for the resistance to radiotherapy. It is well demonstrated that GBMSCs contribute to glioma radioresistance through an increase in their DNA repair capacity [31,32,33,36]. Here, we show for the first time that key DNA damage repair players involved in homologous recombination, which is the repair pathway preferentially used by GBMSCs, can be downregulated by blocking ALKBH5, leading to GBMSC radiosensitization. This major finding highlights the potential of ALKBH5 as a therapeutic target in the treatment of GBM radioresistance since inhibiting this RNA demethylase can block multiple partners of the homologous recombination, including the key players of this pathway, RAD51 and CHK1. 

Although several papers have reported that targeting RAD51 in GBMSCs using inhibitors or siRNA increased the radiosensitivity of GBMSCs [33,36,37,48,49], our study is the first demonstrating that blocking ALKBH5 expression in GBMSCs decreased both the basal and IR-induced expression of RAD51, and inhibited the ability of GBMSCs to repair DNA damages in response to IR, leading to radiosensitization of the cells. In addition to RAD51, we observed several other DNA repair genes induced by IR and involved in HR that were also downregulated by targeting ALKBH5 in GBMSCs. We observed a decrease in the BRCA2, BRIP1, EXO1, and XRCC2, 4 genes, whose high expression in GBM has been correlated with poor patient survival prognosis [50,51]. We also validated the checkpoint kinase 1 (CHK1) involved upstream of the HR pathway, as a target of ALKBH5. CHK1 has also been shown to be upregulated in GBMSCs and preferentially activated following IR. Inhibition of CHK1 increased IR-induced DNA damages and radiosensitivity of GBMSCs [4,52]. 

The results of our study are in accordance with the pro-tumoral role previously reported for m6A mRNA demethylases and demonstrate that ALKBH5 overexpression in GBMSCs may contribute to the efficiency of DNA damage repair, leading to radioresistance. 

The role of RNA demethylases in radioresistance of cancer cells suggests that co-administration of RNA demethylases inhibitors with radiotherapy may offer a potential therapeutic approach to overcome GBM radioresistance. Several RNA demethylases inhibitors have already been developed with a potent anticancer effect. More particularly, FTO inhibitors, meclofenamic acid (MA), or its ethyl ester form (MA2) have been recently identified as specific FTO inhibitors that have shown antitumor activity on myeloid leukemia (AML) and GBM, prostate, and uterine cervical cancers [12,53,54,55,56]. R-2-hydroxyglutarate (R-2HG), an oncometabolite resulting from the conversion of α-ketoglutarate by mutant IDH1/2 enzymes, has also been shown to specifically inhibit FTO and proliferation of leukemic or GBM cells [13]. More recently, a study also reported the efficacy of a specific ALKBH5 inhibitor, ALK04, in reducing tumor growth of melanoma cells [57]. Several other compounds, including (rhein, MO-I-500, Entacapone, NCDPCB, CHTB), have also been described as FTO or ALKBH5 potential inhibitors, although their anti-tumoral action has not been demonstrated [58]. Interestingly, Malacrida et al. recently reported that a new sodium channel blocker inhibited the migration of the GBM cell line U87. Although they do not demonstrate the role of ALKBH5 in the migration of the U87 cells, they showed an off-target effect of this sodium channel blocker on ALKBH5, suggesting that this compound may represent a new potential ALKBH5 inhibitor [59].

In addition to its role in radioresistance, we also demonstrated in this study that ALKBH5 contributes to the aggressiveness of GBM by favoring the invasion of GBMSCs. Indeed, GBMSC spheroids deficient for ALKBH5 exhibited a significant reduced invasion capability relative to control spheroids. This is the first report showing that ALKBH5 promotes GBMSC invasion. Our results emphasize the importance of ALKBH5 as a target to efficiently reduce GBMSC invasion. Indeed, blocking ALKBH5, which regulates the expression of both YAP1 and FOXM1, two transcription factors overexpressed in GBMSCs and previously involved in invasion, might have a higher impact on GBMSC invasion than blocking these two factors independently. In addition, the involvement of ALKBH5 in the invasive capability of GBM cells highlighted by our results is reinforced by the analysis of the TCGA database, which showed, associated with ALKBH5 high expression, several molecular signatures related to cell motility, adhesion, or extracellular matrix organization. These data suggest that in addition to YAP1 or FOXM1 that might contribute to ALKBH5-mediated invasion, numerous other factors related to cell invasion may be involved. 

These results are in accordance with previous reports, which showed that the two m6A mRNA demethylases, ALKBH5 and FTO, promote cell proliferation and invasion in breast, gastric, and ovarian cancers [20,21,60,61]. However, it seems that ALKBH5 and FTO can have an opposite role in other cancer types, suggesting a complex regulation system. In particular, these demethylases suppress malignancy and invasion of pancreatic and colorectal cancers [24,25]. In addition, controversial results have been published in lung cancers. Whereas Jin et al. showed that ALKBH5 expression decreases tumor growth and metastasis by reducing YAP expression [62], other authors reported an increase in proliferation and invasion of lung cancer cells mediated by ALKBH5 or FTO [18,19,22].

In summary, our study highlights the crucial role of m6A mRNA demethylases in regulating radioresistance of GBM. Our results demonstrate that ALKBH5 inhibition, by decreasing HR, which is preferentially used by GBMSCs, might be a novel approach to radiosensitize GBMSC. In addition, we also showed that ALKBH5 might be an attractive therapeutic target to overcome invasiveness of GBMSCs.

## 4. Materials and Methods 

### 4.1. GBM Patient-Derived Cells

GBM biopsy specimens were obtained from the Neurosurgery Department at Toulouse University Hospital as part of a clinical protocol (PI Pr. E. Cohen-Jonathan-Moyal) approved by the Human Research Ethics Committee (ethical code 12TETE01, ID-RCB number 2012-A00585-38, date of approval: 07-05-2012). Written informed consents were obtained from all the patients. Tumors were classified as GBM according to the WHO. All GBM used in this study were IDH1wt. The primary neurospheres were obtained from GBM specimens as described by Avril et al [63] and maintained in DMEM-F12 (GIBCO) supplemented with B27 and N2 (LifeTechnologies), FGF-2, and EGF (Peprotech) at 37 °C in a 5% CO2 incubator. Neurospheres were used during less than 12 passages to keep cell characteristics. The GBMSCs used in the study have been previously characterized [27,28,29,30].

### 4.2. Cells Irradiation

Cells were exposed to different doses of IR, as indicated, using an irradiator XRad Smart plus (Actemium NDT- Products and Systems, Le Plessy, France).

### 4.3. SiRNA Transfection, RNA Extraction, Reverse Transcription, and Real-Time PCR

The siRNA against ALKBH5(1) and YAP1 or the scramble control were purchased from Qiagen (Courtaboeuf, France) and the siRNA ALKBH5(2) was purchased from Ambion (Thermo Fisher, Illkirch, France). Transfection was performed with Lipofectamine RNAi Max (Invitrogen, Thermo Fisher, Illkirch, France) following the manufacturer’s protocol. The RNeasy RNA isolation Kit (Qiagen, Courtaboeuf, France) was used to extract total RNA. Reverse transcription was performed using the Prime Script RT Reagent kit (TAKARA, St Germain en Laye, France). Real-time PCR was performed using the ABI-Stepone+ (Applied Biosystems, Thermo Fisher, Illkirch, France). Normalization was done with GAPDH.

### 4.4. Three-Dimensional Clonogenic Assay

Primary neurospheres from different patients expressing a specific siRNA (si-ALKBH5) or a scramble control (si-Scr) were seeded in 96-well plates (100 cells/wells, 12 wells per condition). After 24 h, cells were treated or not with different doses of gamma rays (2 to 6 Gy). Then, 8–10 days post-IR, the number of neurospheres/well with more than 50 cells was measured. The surviving fraction (SF) was calculated taking into account the plating efficiency in the non-irradiated condition (PE = spheres number/seeded cells number × 100). 

### 4.5. Western Blot Analysis

Proteins, separated by SDS-PAGE were analyzed by Western blot using the indicated antibodies: Actin, ALKBH5 (Millipore, Molsheim, France), Rad51, FOXM1, YAP1, CHK1, phospho-CHK1 (Cell Signaling, Ozyme, St Cyr l’ecole, France), and gamma-H2AX (BioLegend, Ozyme, St Cyr l’ecole, France). 

### 4.6. Three-Dimensional Tumor Spheroid Invasion Assay

Three-dimensional tumor spheroid invasion assays were performed as described by Vinci M. et al. [64]. Briefly, 1000 to 2000 cells were seeded into ultra-low attachment 96-well round-bottom plates to allow formation of 3-D spheroid. Then, 72 h later, the spheroid were transfected with specific siRNA or a scramble control using Lipofectamine RNAi Max (Invitrogen, Thermo Fisher, Illkirch, France) following the manufacturer’s protocol. Then, 24 h after, the transfection half of the growth medium was removed and replaced by the Matrigel. In the experiments with Verteporfin, 72 h after spheroid formation, the inhibitor was added to the medium just before addition of the Matrigel. After 1 h at 37 °C, when the Matrigel was solidified, growth medium was added to the wells. Images were taken at T0 and T24 h using a Nikon microscope and the Nikon software NIS Elements. The spheres area and the area covered by the invading cells was measured using the Image J software.

### 4.7. Genes Correlations

The correlations between ALKBH5 expression and CHK1 or YAP1 were obtained by the co-expression analysis in the TCGA database. Links between biomarkers were assessed using Spearman’s rank correlation coefficients and their associated *p*-values. ALKBH5 expression was plotted on the x-axis, and the expression levels of other genes of interest were represented on the y-axis.

### 4.8. Molecular Signatures Associated with ALKBH5 Expression

The quantile normalized RNAseq gene expression of the glioblastomas cohort (GBM) was downloaded from TCGA. Only samples (*n* = 442) from G4 stage [65] were kept. Based on their ALKBH5 expression, the two groups of samples with the 25% lowest and the 25% highest expression were selected and defined (*n* = 220). The differential gene expression was tested using Wilcoxon or T-test after a normality test [66] and *p*-values were corrected using the Benjamini–Hochberg method [67]. Genes upregulated in the ALKBH5-high group with a log2 Fold Change > 0.5 were selected and compared to the database C5 [68] using Autocompare ZE with Zelen exact (ZE) *p*-value calculation [66,69]. 

## 5. Conclusions

The notion that m6A RNA methylation is an important process in the regulation of key genes involved in carcinogenesis is very recent. This epigenetic modification modulates mRNA expression, leading to pro- or anti-oncogenic effects. In GBM, demethylases are overexpressed, promoting GBMSC growth. In the present study, we showed that demethylases affect other important aspects of GBM development and recurrence: radioresistance and invasion of GBMSCs, suggesting that these enzymes might represent interesting targets in the treatment of GBM.

## Data Availability

The data presented in this study are available on request from the corresponding author.

## Supplementary Materials

The following are available online at https://www.mdpi.com/2072-6694/13/1/40/s1, Figure S1: Checking of a potential off-target effect with ALKBH5 siRNA. Methylases/demethylases expression in GBMSC. 3D invasion using YAP1 siRNA. Figure S2: Original western blot gels for Figure 1B. Figure S3: Original western blot gels for Figure 2B. Figure S4: Original western blot gels for Figure 4A,B. Figure S5: Original western blot gels for Figure 4C. Figure S6: Original western blot gels for Figure 6C. Table S1: Molecular signatures associated with high ALKBH5 expression in TCGA database.
