# Supplementary material for: The m6A RNA Demethylase ALKBH5 Promotes Radioresistance and Invasion Capability of Glioma Stem Cells
Source: Cancers (Basel). 2020 Dec 25;13(1):40. doi: 10.3390/cancers13010040 (PMC7795604; doi:10.3390/cancers13010040)
Supplement: Supplementary file 1 [file cancers-13-00040-s001.zip › cancers-1025874-supplementary.pdf]

The m6A RNA Demethylase ALKBH5 Promotes Radioresistance and Invasion Capability of Glioma Stem Cells

Aline Kowalski-Chauvel, Marie Géraldine Lacore, Florent Arnauduc, Caroline Delmas, Christine Toulas, Elizabeth Cohen-Jonathan-Moyal and Catherine Seva

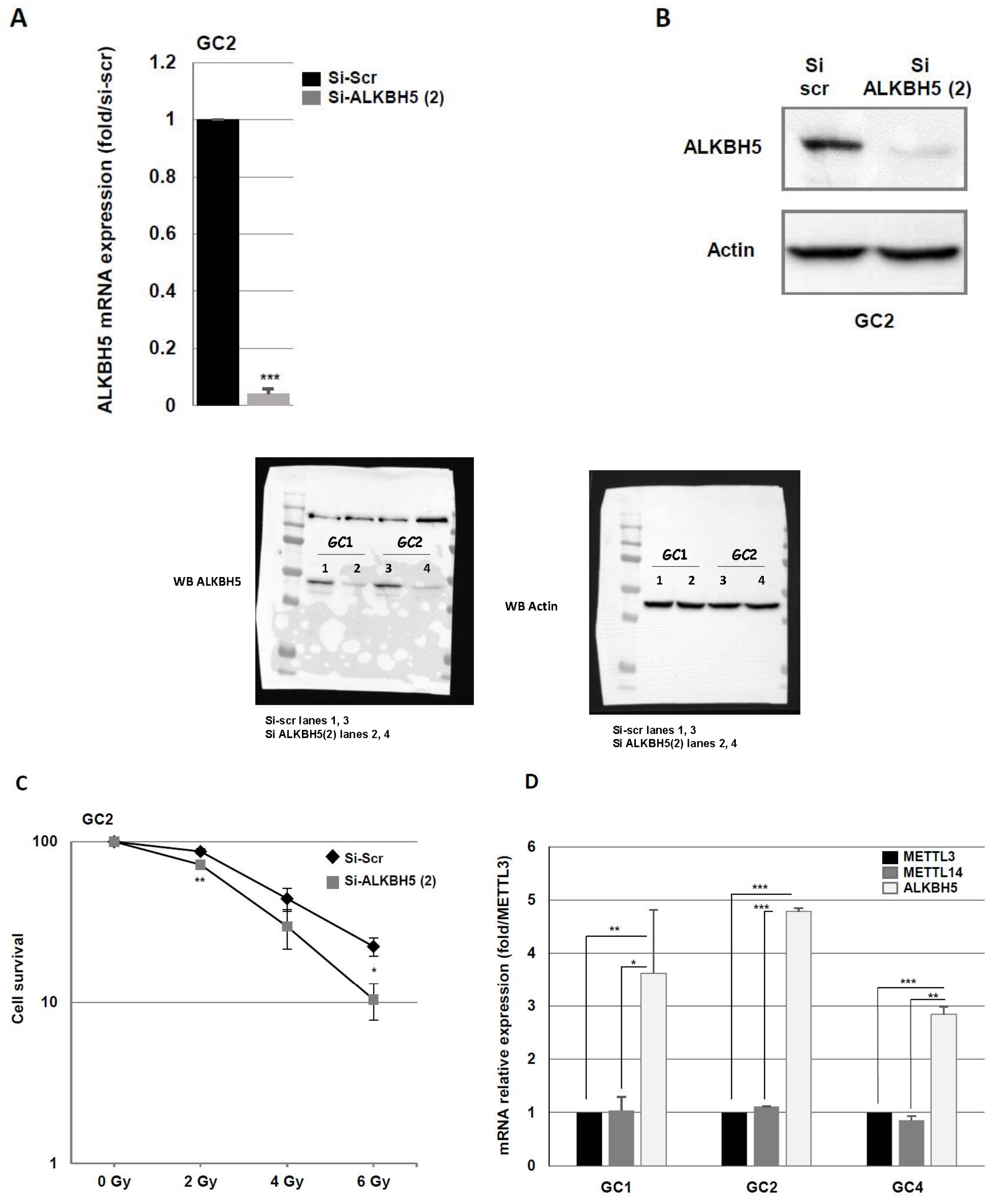

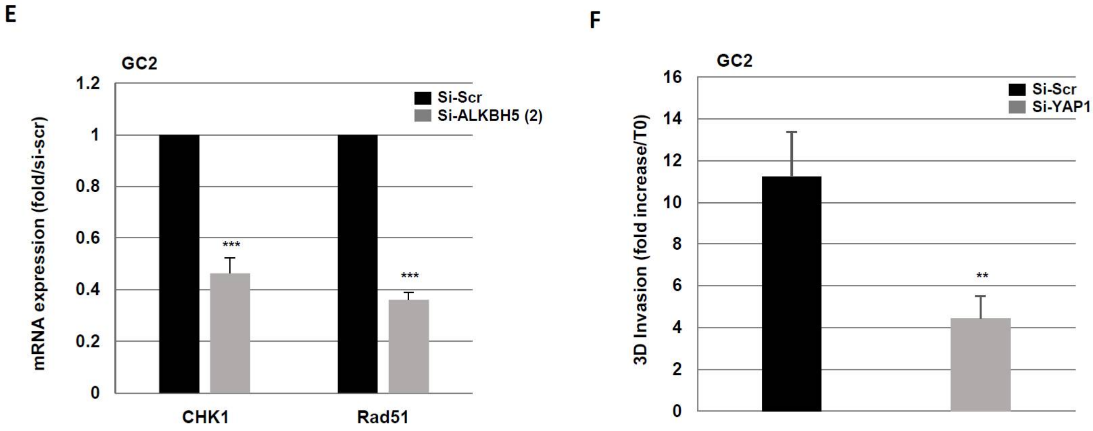

Figure S1. (**A**–**E**) Checking of a potential off target effect with ALKBH5 siRNA. GC2 primary neurospheres were transfected with a specific siRNA (si-ALKBH5 (2)) or a scramble control (si-Scr) as indicated. ALKBH5, CHK1 or Rad51 expression was analyzed by (**A**,**E**) real time PCR or (**B**) western blot. (**C**) Cells were analyzed in 3D clonogenic assays as described in "Methods". (**D**) Methylases/Demethylases expression in GBMSC. METTL3/METTL14 and ALKBH5 expresion was analyzed by real time PCR in primary neurospheres from 3 different patients (GC1, GC2, GC4). (**F**) 3D invasion using YAP1 siRNA. Quantification of 3D tumor cells invasion was performed with GC2 primary neurospheres as described in "Methods". Quantifications of 3 expriments are presented as means ±SD. ***$p < 0.001$; **$0.001 < p < 0.01$; *$0.01 < p < 0.05$.

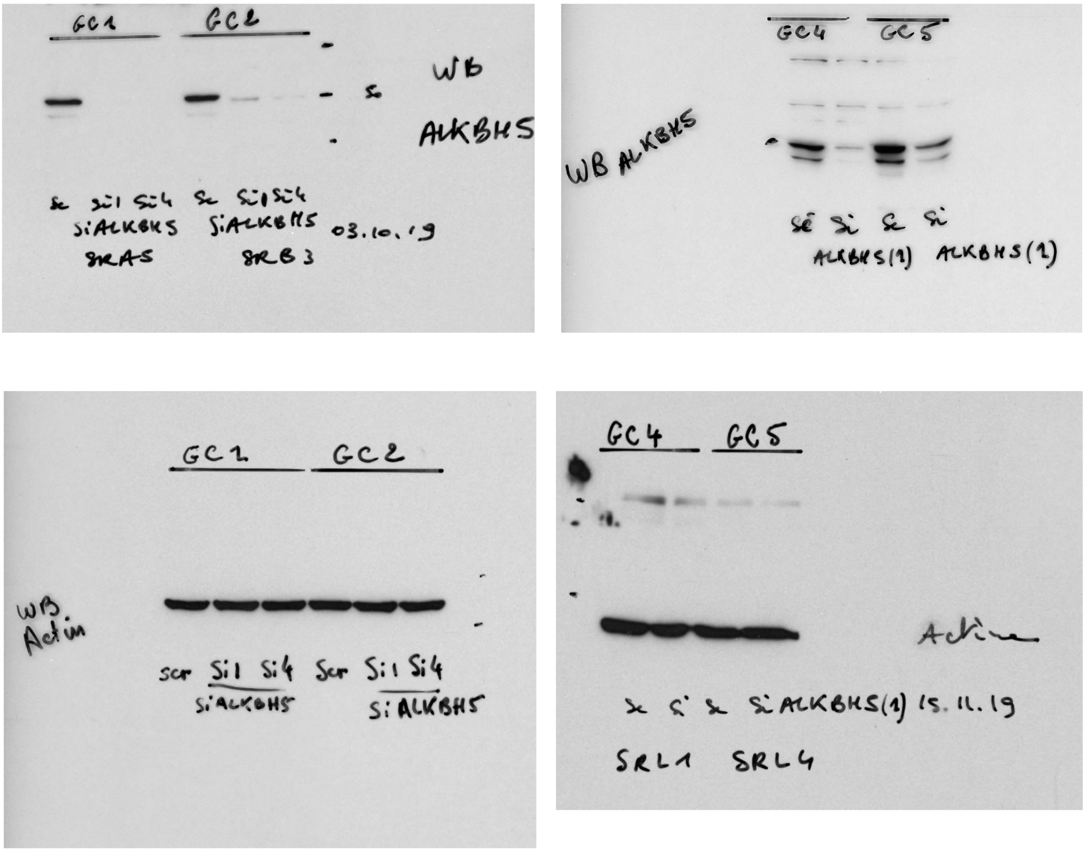

Figure S2. Original western blot gels for Figure 1B.

GC1

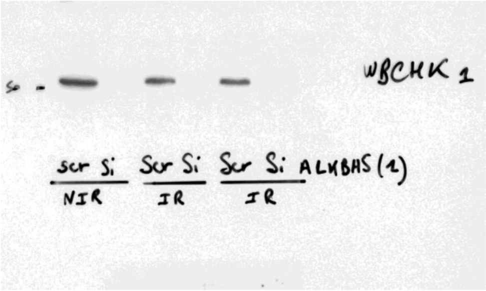

GC2

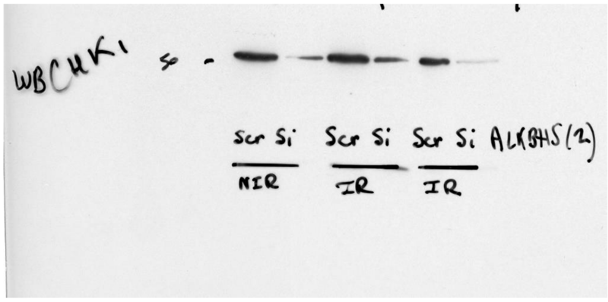

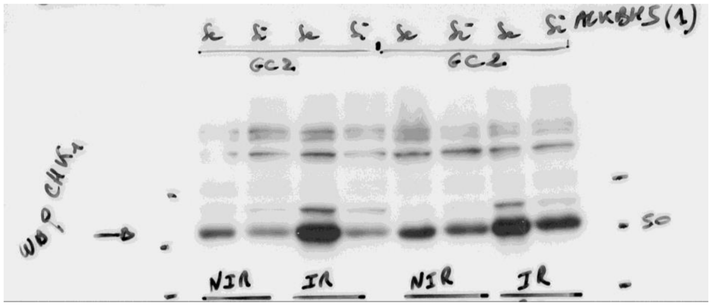

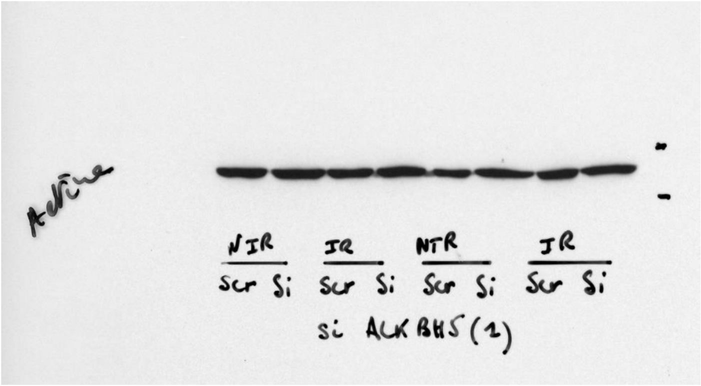

Figure S3. Original western blot gels for Figure 2B.

Original gels for Figure 4A

GC1

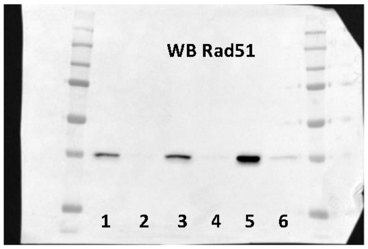

Si-scr lanes 1, 3, 5
Si ALKBH5(1) lanes 2, 4, 6
NIR lanes 1, 2
IR 3h lanes 3, 4
IR 24h lanes 5, 6

GC2

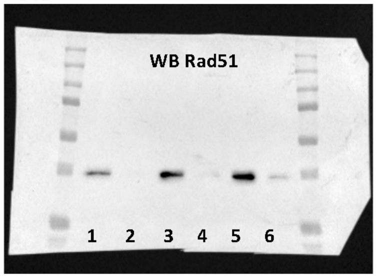

Si-scr lanes 1, 3, 5
Si ALKBH5(1) lanes 2, 4, 6
NIR lanes 1, 2
IR 3h lanes 3, 4
IR 24h lanes 5, 6

GC1

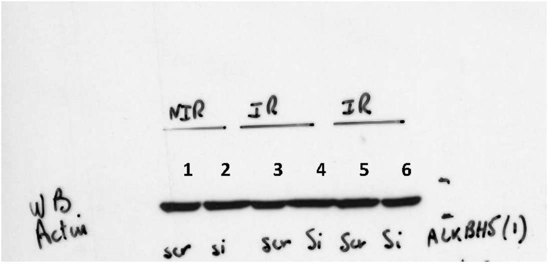

GC2

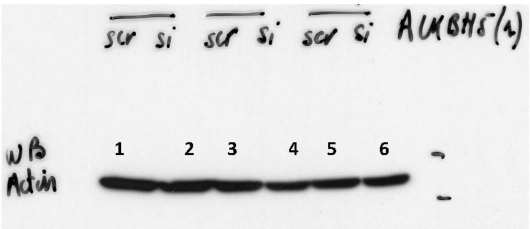

Original gels for Figure 4B

GC1

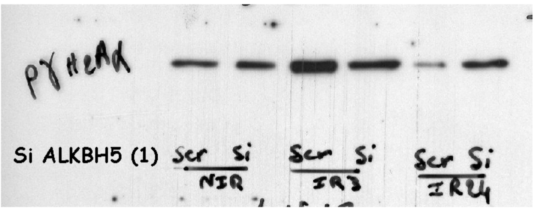

GC2

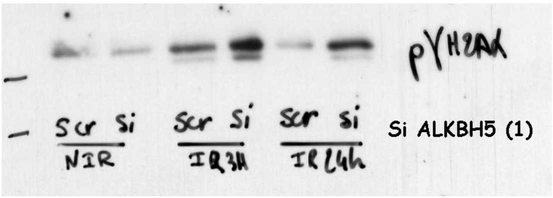

GC1

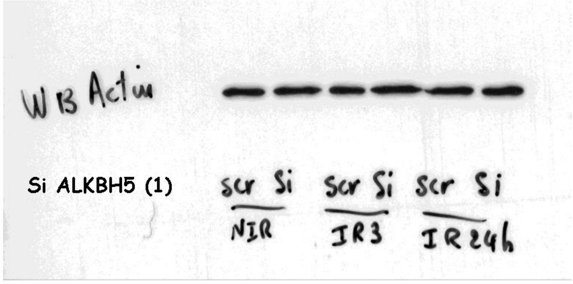

GC2

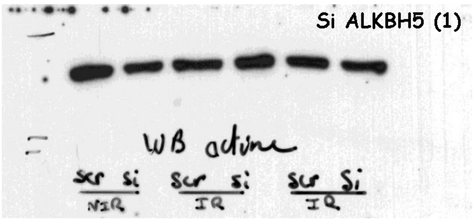

Figure S4. Original western blot gels for Figures 4A,B.

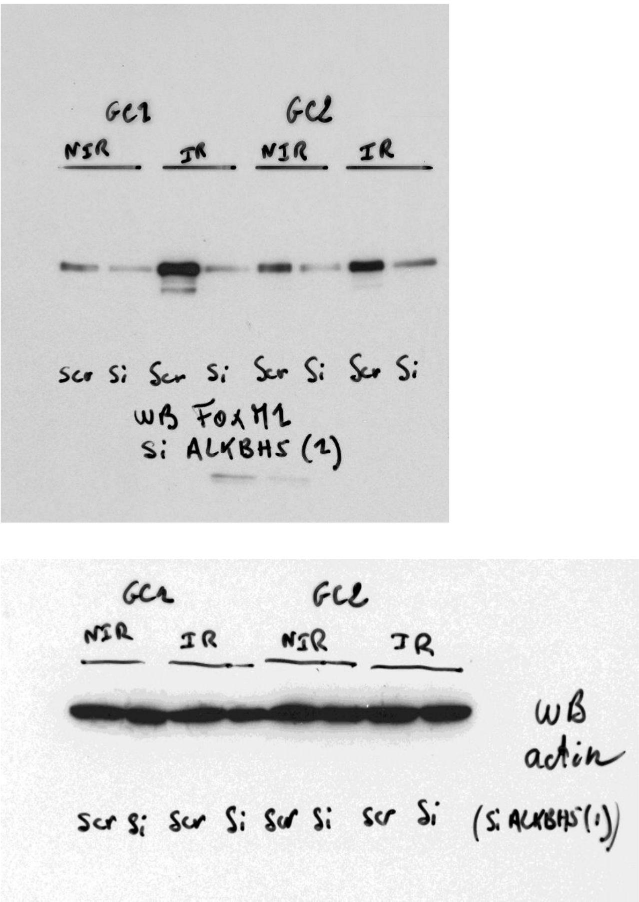

Figure S5. Original western blot gels for Figure 4C.

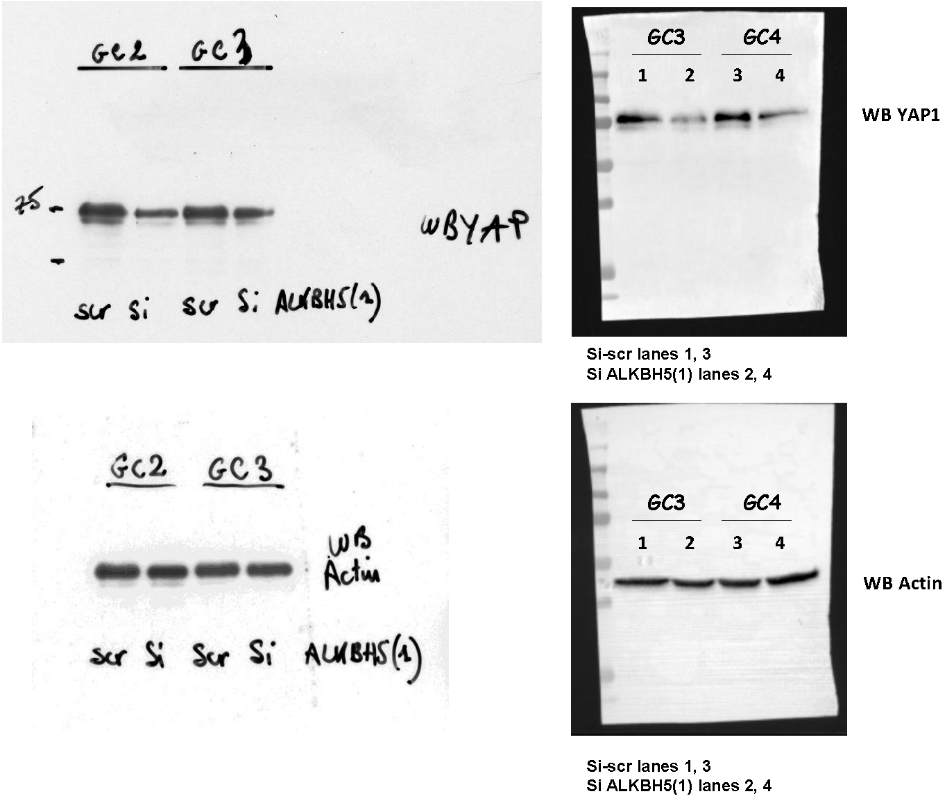

Figure S6. Original western blot gels for Figure 6C.

Copyright: © 2020 by the authors. Licensee MDPI, Basel, Switzerland. This article is an open access article distributed under the terms and conditions of the Creative Commons Attribution (CC BY) license (http://creativecommons.org/licenses/by/4.0/).
